# Emotional Competence in Primary School Children: Examining the Effect of a Psycho-Educational Group Intervention: A Pilot Prospective Study

**DOI:** 10.3390/ijerph19137628

**Published:** 2022-06-22

**Authors:** Sabina La Grutta, Maria Stella Epifanio, Marco Andrea Piombo, Pietro Alfano, Agata Maltese, Salvatore Marcantonio, Sonia Ingoglia, Marianna Alesi, Rosa Lo Baido, Giacomo Mancini, Federica Andrei

**Affiliations:** 1 Department of Psychology, Educational Science and Human Movement, University of Palermo, 90128 Palermo, Italy; sabina.lagrutta@unipa.it (S.L.G.); mariastella.epifanio@unipa.it (M.S.E.); agata.maltese@unipa.it (A.M.); sonia.ingoglia@unipa.it (S.I.); marianna.alesi@unipa.it (M.A.); 2 Department of Psychology “Renzo Canestrari”, Alma Mater Studiorum-University of Bologna, 40127 Bologna, Italy; marcoandrea.piombo2@unibo.it (M.A.P.); federica.andrei2@unibo.it (F.A.); 3 Institute of Traslational Pharmacology (IFT), National Research Council of Italy, 90146 Palermo, Italy; 4 Quality, Planning and Strategic Support Area, University of Palermo, Piazza Marina 61, 90133 Palermo, Italy; salvatore.marcantonio@unipa.it; 5 Department of Biomedicine, Neuroscience and Advanced Diagnostics (Bi.N.D.), University of Palermo, 90129 Palermo, Italy; rosa.lobaido@unipa.it; 6 Department of Education Studies “Giovanni Maria Bertin”, Alma Mater Studiorum-University of Bologna, 40126 Bologna, Italy; giacomo.mancini7@unibo.it

**Keywords:** emotional competence, emotional development, emotional skills psycho-educational intervention, primary school children, group intervention

## Abstract

Emotional competence (EC) is a key component of children’s psychological, cognitive, and social development, and it is a central element of learning. The primary goal of this study was to evaluate the effectiveness of implementing a psycho-educational group intervention aimed at improving children’s emotional competence (EC), quality of integration and scholastic skills. A total of 229 children (123 females; M Age = 7.22 years; SD = 0.97 years) completed the Pictures of Facial Affect (POFA), the Drawn Stories Technique, the Classroom Drawing, and the Colored Progressive Matrices. The total sample was randomly divided into an intervention group (N = 116) who took part in psycho-educational activities and a control (no-intervention) group (N = 84). Both groups were tested at baseline, before the intervention started, and at the end of the intervention (4 months from baseline). Results from mixed-model ANOVA revealed a significant main effect for POFA score over time (F = 6.24, *p* = 0.01) and an interaction effect between POFA and group (F = 4.82, *p* = 0.03). No significant main effect was found for classroom drawing over time (F = 0.81, *p* > 0.05) or for quality of integration and group intervention. These findings support the importance of developing psycho-educational programmes in school for promotion of emotional health for preventing not only the onset of problematic behaviours at school such as bullying but also the development of clinical conditions linked to difficulties in emotional recognition, expression, and regulation such as alexithymia.

## 1. Introduction

School is the primary context where children develop their knowledge, thinking, and attitudes. These essential attributes are impregnated by affectivity, which acts as a psychological function regulating a personality’s healthy development and a feeling of authenticity and critical thinking [1]. School is also an institution made up of group sets: Everything takes place in a group format, and the classroom environment can be considered a special group. For these reasons, the development of emotional competence can be considered a central element of learning [1]. Emotional competence (EC) refers to a set of skills at identifying, understanding, and responding to one’s own emotions and those of others [2]. Saarni [3] suggests that EC is made up of eight different basic abilities: (1) awareness of one’s own emotions; (2) ability to recognize others’ emotions; (3) ability to use the vocabulary of emotion and expressions; (4) ability to empathize with others’ emotional experiences; (5) ability to differentiate subjective and internal emotional experience from external, emotional expression; (6) capacity for adaptive coping with aversive emotions and distressing circumstances; (7) awareness of emotional communication within relationships; and (8) capacity for emotional self-efficacy [3]. Among these skills, the ability to recognize emotions is fundamental to managing and regulating emotions, permitting children who decode emotions well to reach higher academic achievement and to be better adjusted personally and socially [3,4]. The understanding of emotions, as a complex ability, gradually develops from preschool to school years [5] according to three hierarchical periods: at five, children understand the public elements of emotions and their expressions; at about seven, they recognize the nature of emotions in terms of expressed and underlying emotions; and at about nine, they comprehend reflexive connotations of emotion including knowledge to regulate emotions [6]. Some studies underline that EC and its related emotional abilities cannot be separated from individuals’ social skills and functioning, aimed at achieving better performance in social situations [7]. These qualities can be acquired through learning and interpersonal and social interactions, especially in children [2].

In particular, groups can enhance learning through shared experiences, gaining a sense of belonging and practising their skills within a safe, contained space with adults who are available to provide support [8]. Schools, and classrooms in particular, are often the primary context where children accept or refuse experiences with their peers [9]. Promoting healthy social and emotional development can help children to manage their needs in relation to their social and educational environments, to keep good relationships with peers, to recover from negative emotions, to tolerate frustrations, and to express emotions in adaptive manners [10]. These relationships not only help to modify cognitive and emotional development but also are crucial components of scholastic well-being as they provide indications of children’s adaptation in their classrooms [11,12]. Moreover, several studies attest the important role of emotional competences in psycho-educational processes during childhood as well as their relevance for the development of social competence in future years [13,14].

To the best of our knowledge, the literature has often overlooked the efficacy of group interventions with children. However, in the last few years this area of investigation has gained more popularity, and several studies have been published on topics including children’s group treatment outcomes [15], mindfulness-based interventions [16], the effectiveness of resilience-based interventions [17], and school problem prevention [18]. Evidence on school prevention programmes pertains to children’s peer relationships, emotional and behavioural problems [19,20], social anxiety, [21], bullying, victimization [22], delinquency [23], self-esteem, social skills [24], and aggressive behaviour [25]. Specifically, Schechtman and Ifargan [26] suggested that psycho-educational groups are most effective with primary school children, serving as a tool to improve children’s awareness of their behaviour, find what triggers their behaviour, and learn how to control it [27]. Moreover, meta-analytic research findings on psychotherapy with this age group [28] indicate effect sizes which are comparable with those for empirically based adult treatments, which have emerged as well-established with children and adolescents [20]. Promoting healthy social and emotional development can help children to manage their social and educational needs in their daily environments, to keep good relationships with peers, to recover from negative emotions, to tolerate frustrations, and to express emotions in adaptive manners [10]. These relationships not only help to modify cognitive and emotional development but also are crucial components of scholastic well-being as they provide indications of children’s adaptation in their classroom [11,12].

### The Current Study

The present pilot study is part of a larger prospective intervention research involving several Italian primary schools. Specifically, it focuses on the first year of a three-year group intervention programme, aimed at enhancing different aspects of children’s EC. The main aim of the current study was to explore the effect of a group psycho-educational intervention on children’s ability to recognize emotions and scholastic integration in the primary school context. The following hypotheses were formulated:

(1) The quality of school integration decreases along the scholastic path in the absence of a psycho-educational intervention based on development of emotional skills.
(2) The ability to recognize emotions is correlated with the quality of integration and both have positive effects on cognitive nonverbal ability.
(3) A psycho-educational intervention based on the empowerment of emotional competence and promotion of integration can improve children’s skills meaningfully.

This study is designed as a pilot prospective study.

## 2. Materials and Methods

### 2.1. Study Design

This pilot study is designed as a prospective study, and the selection of schools was based on previous work relationships with schools in order to collect a convenience sample. Children were eligible for the study if they were between 7 and 8 years and belonged to the third class of two primary schools in the city of Palermo, so they could participate ideally in at least three intervention years. Class groups were randomly assigned to either the intervention (four class groups, N = 138) or the control (four class groups, N = 91) condition. Specifically, group assignment was conducted following the letter indicating class sections: Sections A and B in each school were included in the intervention group, while sections C and D in each school were included in the control group. Children in the control group were not informed that there were other classes in which intervention was undertaken. Tests were presented to the children as activities that would be repeated twice during the academic year, and they were administered by the two researchers who undertook the intervention. This study was approved by the Ethics Committee of the University of Palermo (no. 83/2022).

### 2.2. Participants

Parental permission was obtained for 229 children (n = 123 females), aged 7 to 8 years (M = 7.22 years, SD = 0.97), attending eight different classes at schools located in a middle-class area of the city of Palermo, Italy. No children were withdrawn from the study by parents. Of the 229 participants, 200 provided both Time 1 and Time 2 data on the variable measures. Of the 29 who failed to provide complete data, 7 were missing both the Time 1 and Time 2 data, 5 were missing Time 1 data but turned in Time 2 data, and 24 turned in Time 1 data but were missing Time 2 data. Missing data did not reflect participant dropout but referred to students who completed the program but did not complete the selected measures due to being absent on the day of data collection. Hence, complete data were collected from 200 children.

### 2.3. Measures

The Drawn Stories Technique [29]. The Drawn Stories Technique is a graphic technique aimed at evaluating children’s current emotional state. The Drawn Stories Technique asks a child to draw on a sheet of paper a story invented by the child. The psychologist does not insist on any point of view and waits until the child has drawn the story. Through this technique, children can express their affective themes and internal conflicts. These stories can be classified in four different ways depending how the story ends: (1) Positive Outcome (PO), when the narration ends positively without any accident; (2) Negative Outcome (NO), when the narration ends negatively with an accident; (3) Compensated Positive Outcome, when despite the presence of an accident, the narration ends positively; (4) Absent Outcome (AO), when the story is not completed. After the drawing phase, children are asked to write the story behind the sheet and then they are briefly interviewed by the researcher about their stories. The aim of this interview is to determine which character the child identifies with in the story. After that, it is possible to score the type of outcome based on what happens to the chosen character. In particular, positive outcomes (PO and CPO) are emotional well-being indicators, while negative outcomes (NO) and absent outcomes (AO) are indicators of emotional turbulence and as a block of symbolic expression [30]. The drawings are scored by the researcher and one other expert in the field to reach a common evaluation in case of doubt, with good interrater reliability (r = 0.80).

The Classroom Drawing [31]. The Classroom Drawing is a graphic technique that explores the quality of scholastic integration perceived by children and their relationships with teacher and classmates. Classroom integration is evaluated as an index ranging from 0 to 3. In particular, 1 point is calculated for the presence of the teacher in the drawing (relationship with authority), 1 point for the presence of classmates (level of socialization) and 1 point for the presence of the drawer himself (personal involvement in the class). In practice, the scores indicate the following: 0 = worst integration (no people are present in the drawing) to 3 = best integration (every element is present in the drawing).

Picture of Facial Affects [32]. The Picture of Facial Affects is composed of 110 black and white photos in which some actors reproduce six basic emotions (anger, sadness, fear, happiness, surprise, disgust). In this study, 2 series of 14 photos were selected, only with female faces because primary school children are more likely to interact with females (e.g., mothers or teachers), and this could facilitate emotional recognition. After the presentation of the photos, the children had to write on a sheet the correct emotion for every item. Neutral items were not calculated in the total. The score ranges from 0 to 12, with 1 point for each correct answer. These two series of photos were used to evaluate children’s emotional facial recognition abilities.

The Diagnostic Analysis of Nonverbal Accuracy 2-Receptive Posture (DANVA-2 POS) [33]. The DANVA-2 POS was administrated to evaluate non-facial emotion recognition ability by posture presentation. It consists of 24 photographs of an equal number of happy, sad, angry, and fearful emotions of high and low intensities in both standing and seated postures. The experimenters used a black permanent marker to cover up the facial expression of the model in each photograph. There are three different scores: a score that is the sum of all the correct answers about emotions, another score that is the sum of all correct answers about the intensity of emotions (high or low), and a comprehensive score that is the sum of all the times when both emotions and intensity were correctly answered. This test showed good internal consistency with a Cronbach’s coefficient alpha of 0.68 and good test–retest reliability after a 2-week interval (r = 0.85, *p* < 0.05) [33].

Colored Progressive Matrices [34]. The CPM were used to evaluate the cognitive nonverbal ability of the children; they are suitable for younger children (up to 11 years old). They consist of 36 items presented in 3 sets of 12. Each item requires completing a series of figures with what is missing, compared with a model, according to increasing difficulty. The subject has to understand the underlying logic and apply it to reach the solution. The last Italian validation of the test showed optimal internal consistency with a Cronbach’s coefficient alpha of 0.88 and reliability = 0.85 [35].

### 2.4. Procedures

At the beginning of the school year (2017–2018), school principals, teachers, and parents were informed about the purposes of the research and the main features of the emotion-based educational program. Written consent was signed and collected. Data collection was conducted twice, and measures were administered to the group the first time before the intervention in January (T1) and the second time after the intervention at the end of the school year in May (T2). The study was approved by the Bioethics Committee of the University of Palermo (n. 83/2022).

#### Intervention

The intervention was provided to 4 classes out of 8 during an academic year. It was based on socio-emotional education methodology in a group setting, and it was structured in 10 weekly meetings at school, lasting 2 h, within the normal class schedule from March to May. It was structured in a series of semi-operative activities, as drawings or teaching sheets, in order to develop children’s emotional competence and classroom integration. The teacher, one conductor, and two attending observers were present at every meeting with the children. These meetings were structured in two different steps: the children presenting and carrying out the activities and the children showing and sharing their own works and discussing them in group. The intervention specifically was conceived over a period of 4 consecutive years and was structured in different levels following the same methodology and structure as previously discussed.

The first intervention year consisted of some activities intended to promote the children’s emotional alphabetization, that is, their capacity to recognize their own and others’ emotions and regulate and express them in an adaptive way. Every single activity was conceived to develop various emotional competence dimensions: self-perception, emotion recognition and expression skills (using different types of expressions like photographs, colours, drawings, words), learning different thinking modalities (creative, descriptive, emotional, optimistic, pessimistic), and integrating them to find better problem-solving solutions. Specifically, in the first and second meeting, children were asked, “If you were an animal, what animal would you want to be?” They were to draw their character on a sheet and include it in an invented story shared with their classmates. These two activities were focused on self-perception and on recognizing one’s own character in the story focusing on emotional components of the storytelling. The third and fourth meetings were aimed at teaching how to express emotions using colours. In particular, children were asked to make a link between colours and emotions on a specific sheet on which they had to colour a drawn colour tube and write the related emotion in their opinion. After that, they talked about the reasons why they chose their association in a group and expressed their “here and now” emotions using colour tubes to make a painting. The fifth meeting was about recognizing and expressing emotion bodily with facial expression. The children were asked to express the seven basic emotions with facial expressions during a class group looking at a photo without being provided with any clues. In the sixth meeting, the children were asked to answer the following question, “What do I think about my class?”, using different thinking modalities. Specifically, six hats in different colours were randomly distributed to the children to wear, and each hat colour represented one thinking modality: red = emotional thinking, black = pessimistic, yellow = optimistic, green = creative, white = descriptive, and blue = complex thinking, which integrated all the previous modalities. The children had to express their thoughts on the basis of the hat that they wore and discuss the issue together. The seventh meeting was focused on thinking about trust in oneself and others. Specifically, the children were asked to imagine being a circus acrobat at the top of a human pyramid and indicating on a sheet on which acrobats are drawn which people in their lives supported them to prevent them from falling. The eighth meeting was focused on acquiring awareness of the emotions experienced within their different social environments: “We have travelled by sea… where has our boat landed?” Three different islands were drawn on a poster, Friend Island, Classmates Island, and Family Island, and the children were asked to put some paper boats on one or more islands discussing these choices in a group. The ninth meeting was focused on improving the ability to reflect on different aspects of the “journey”. The children were asked to answer the following question: “The journey is about to end, what do you put in your bag?” A big paper bag was created, and the children were asked to write their thoughts, feelings, expectations, hopes, or worries in it. Finally, the last meeting was structured as a free group discussion in which children had the opportunity to express their satisfaction about the activities they liked or not.

### 2.5. Statistical Analyses

Power analysis was performed with G* Power 3.1 Software in order to determine the minimum total sample size required for this study, and the results showed that at least 102 participants (51 for each group) were needed to register a medium Cohen size effect d = 0.50. Descriptive statistics and frequency analysis were used to investigate the demographic characteristics. We gathered pre- and post-intervention measures at two time-points, at which we assessed cognitive nonverbal ability, emotional facial recognition abilities, and classroom integration. The Pearson chi square (χ^2^) test of significance and measures of association were applied for the verification of the Drawn Stories Technique at baseline and at T1 for the experimental and control groups.

We also measured the emotional states of the children, and we gathered data on their performance at T1 and T2. To test our hypotheses, a series of mixed-model repeated measure analysis of variance (ANOVA) was used. For each model, the global score for each variable considered was set as the outcome variable, while the group (experimental group vs. control group) was considered an in-between factor; assessment time (pre- vs. post-intervention) was a within factor. Statistical analyses were performed using SPSS (version 21) for the MacOs system. On all statistical tests, a *p* value of less than 0.05 was considered significant.

## 3. Results

No differences in age, t (1.67) = 1.15, *p* = 0.26, gender, χ^2^(1.199) = 0.71, *p* = 0.45, were found between the intervention and control groups. The means and standard deviations of the scores, divided by gender, for both experimental and control group children are shown in Table 1.

Descriptive statistics and paired *t*-test for each group were performed (Table 2).

### 3.1. POFA

To assess the changes in the children’s mean scores over time, their data were analysed using a two-way mixed-model analysis of variance (ANOVA) with the group as a between-subjects variable and repeated measures (within-subjects) as the time factor (baseline to post-intervention follow-up). A series of mixed-model ANOVAs were run to discern changes in the participants’ mean scores in the measurement scales used. Levene’s test was run to discern whether the assumption of homogeneity of variances had been violated.

**Figure 1:**
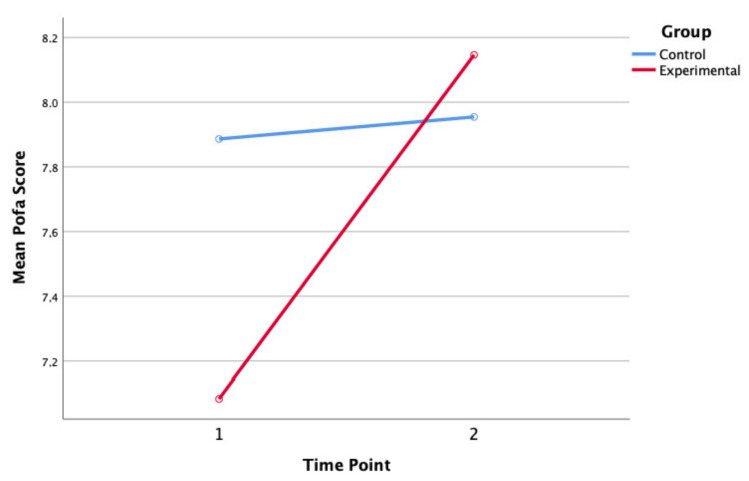
Mean Pictures of Facial Affect (POFA) for experimental and control group children at baseline (1) and post-intervention follow-up (2).

The mixed-model ANOVA revealed a significant main effect for POFA over time (F (1, 199) = 6.24, *p* < 0.01). The interaction between emotional recognition and group was also significant (F (1, 199) = 4.82, *p* < 0.03) (Figure 1). In contrast, the control group children’s emotional symptoms scores increased slightly, but this increase was not significant at the 0.05 level (t (85) = −0.158, *p* > 0.05).

### 3.2. Classroom Drawing

The mixed-model ANOVA revealed no significant main effect of classroom integration index over time (F (1, 199) = 0.33, *p* > 0.05), and neither was there a significant interaction between classroom integration index and group (F (1, 199) = 2.01, *p* > 0.05).

### 3.3. Danva

There was a significant main effect for Danva scores over time (F (1, 199) = 6.26, *p* = 0.01). However, the mixed-model ANOVA did not reveal a significant interaction between intervention group and Danva scores (F (1, 199) = 0.19, *p* > 0.05); inspection of the means suggested that the scores of both groups increased.

### 3.4. CPM

To investigate changes in the children’ mean MPC scores over time, the data were analysed using a two-way mixed-model analysis of variance (ANOVA) with group as a between-subjects variable and repeated measures (within-subjects) on the time factor (baseline to post-intervention follow-up). The mixed-model ANOVA revealed a significant main effect for scores over time (F (1, 199) = 6.44, *p* = 0.01). Furthermore, there was not a significant interaction between scores over time and group (F (1,199) = 4.13, *p* = 0.07) indicating that the scores of both groups increased.

### 3.5. Drawn Stories Technique

To describe the variations among all children on an individual basis for the Drawn Stories Technique, a comparison of pre- and post-intervention for each category is shown (Table 3).

The Pearson chi square (χ^2^) test results demonstrated that the experimental and control groups were equivalent at baseline (*p* = 0.52). Moreover, while no difference was found at T2 for the control group (*p* = 0.71), in the experimental group, we found a significant difference (*p* = 0.004). As previously described, the Drawn Stories Technique has 4 levels that can be used to identify children whose score suggests—emotional well-being and resilience or turbulence. At baseline, the experimental group children showed 33.6% positive outcomes, 9.5% negative outcomes, 55% positive outcomes compensated, and 1.7% absence of outcome. At post-intervention we registered, as shown, minimal changes in the outcome. Concerning the control group, at baseline, 40.6% had a positive outcome, 4% a negative outcome, and 52% a compensated positive outcome. At time 2, we registered much more movement in the outcome: 8% each moved to a negative outcome and a positive outcome, while there were far fewer compensated positive outcomes (16%). An important conclusion can be drawn from this since our study showed that positive outcomes are frequently registered in younger children and compensated positive outcomes become more frequent later while there is an increase of negative outcomes in adolescents and preadolescents, especially males.

## 4. Discussion

The primary goal of this study was to evaluate the effectiveness of a psycho-educational group intervention aimed at improving emotional health, quality of integration, and children’s scholastic skills. Many studies have shown that classroom-based psychological interventions not only have effects in increasing children’s well-being but also in enhancing academic skills and decreasing behavioural problems, which promotes a positive atmosphere in classrooms [36]. As hypothesized, this program significantly improved children’s emotion recognition skills. Indeed, primary schoolchildren who participated in the experimental group showed more positive changes than non-participating controls on a measure of emotional recognition after the first year of intervention. Our data showed a trend-level effect of time for both groups in identifying nonverbal expressions of affect. These findings support previous research demonstrating that recognition of a more complex emotional pattern is a function not only of age but also of good cognitive development [37]. Emotional competence can play a key role in enabling children to use the group as a component of change. Moreover, many studies have shown that children’s understanding of emotions is linked to a propensity to reconcile in conflict situations [38] and is a good predictor of their school adjustment [39] and achievement [10,40]. In addition, children who are more emotionally competent are less likely to experience anxiety, depression, and anger [28,41]. Our data showed a trend-level effect of time for both groups to identify nonverbal expressions of affect.

With respect to cognitive nonverbal ability, the results indicated only an effect of time; we did not find a significant interaction between scores over time and group. Effectively, it seems that there were initially consistent differences between the groups, which could have been related to the randomization and could have been referring to some variables that were not controlled in this study, such as levels of individual development of the group class or teaching style. This rise of intelligence over time, observed in both groups, is commonly known as the Flynn effect and has been linked to many factors such as increased environmental complexity [42] or socialization practice at home and at school [43]. In particular, nonverbal intelligence showed increasing performance with age [44]. Furthermore, the literature suggests that the age between 7 and 9 years represents a stage where children start to realize that emotions can be regulated by cognitive strategies and that moral rules could also have an impact on emotional experiences [45,46,47].

Contrary to our expectation, the quality of integration did not change in the experimental group at the end of the first observation year. This result may be explained by the complexity of the school environment, in which some variables, such as teachers’ educational styles, the physical spaces of schools, and the individual differences in children in a class are difficult to control in the current evaluation [48]. The Classroom Drawing is a measure of the quality of scholastic integration and affective climate of the class and, at the same time, could be an important indicator of pupils’ academic success [49,50]. The Drawn Stories Technique can be considered an adequate test to be used with children together with other measures to detect psychological distress [29]. In the present study, the control group at Time 2 showed more changes in negative, positive, and compensated positive outcomes, proving less stable compared with the experimental group. These preliminary results are consistent with many studies that have shown how a positive affective climate, especially in primary school classes, improves children’s learning experience and academic success, with a significant reduction in behavioural problems and learning disabilities [51,52,53]. Movement between positive outcomes and positive outcomes compensated could be considered normal, and the presence of negative outcomes is not a negative sign on the whole. It indicates a presence of an emotional turbulence in the “here and now”, but the possibility to express it through a drawing is fundamental and is the easiest way that children have to contain and regulate it even when they are unable to express it with words. In this regard, as a clinical consideration, we think that positive outcomes could be considered a simpler or more “primitive” modality for expressing emotional states; subsequently, increases in positive outcomes compensated and negative outcomes could reflect a more complex reality and could be considered an evolutionary advancement reflecting children’s development as they become more capable of both accessing resilience resources and expressing conflictual themes such as aggressiveness or anxiety through drawings. For these reasons, we think that the use of this technique could be the first step toward emotional alphabetization programmes in a psycho-educational context aimed at preventing problems in emotional expression and regulation such as alexithymia. Otherwise, the preliminary results of this study are promising and can be interpreted as support for the effectiveness of the group intervention. Furthermore, group work in an elementary school setting is a useful service for remedial and prevention purposes to address students’ academic, social, and emotional concerns [54,55].

### Limitations

The current study provides a short-term assessment of the beneficial effects of a group psycho-educational intervention. The findings must be considered preliminary and interpreted with caution due to the following factors: (a) the study sample size was small, which compromised the statistical power to detect significant differences, (b) there was a lack of additional sociodemographic data for the children, (c) there was a lack of follow-up of at least 6 months after the conclusion of the intervention, and (d) this was a pilot and exploratory study. Additionally, and not least, the involvement of the researchers as the deliverer of the intervention could have influenced the intervention effect. However, this was inevitable since the efficacy study proceeds to the effectiveness trial in the intervention development, which allows the achieving high implementation fidelity [56]. These factors can be a threat to external validity and may inhibit the generalisability of the results of the study to the wider reference population. External validity is called into question when the results of a study cannot be generalised to a larger population or to similar populations in terms of context, setting, participants, and time [57].

The relationships of cognitive skills, emotional competence, and quality of integration required more observation than just the pre- and post-program measures that were used. The study design we used did not allow for proving if the differences between groups on any of the outcome measures could be due to systematic factors or to chance rather than to the group program. In fact, while it is important to evaluate whether interventions meet set goals, it is also necessary to ensure that any gains achieved in the short term are long-lasting [58]. Therefore, future research needs to address whether the gains identified in this study are maintained over a longer time period, exploring also whether the efficacy of similar interventions could be demonstrated with larger samples [59].

The positive effects of the intervention on emotional recognition could also have been linked to uncontrolled factors. For example, they could have been associated as shown in previous research with increased enjoyment and decreased boredom [60]. Finally, the fact that the teachers for the experimental group were different from the teachers for the control group could have acted as a confounding variable. Future researchers should try to take into account this issue when designing and implementing a randomized control trial. Additionally, sociodemographic data need to be included because cultural determinants continue to be good predictors of the risk of developing a mood disorder over a lifetime. Furthermore, socioeconomic deprivation may be a common pathway through which several sociodemographic and cultural determinants of mood disorders act [61].

In our study, organizational reasons in schools ruled out the possibility of having more control over the assignment of students to the experimental or the control group. However, these findings are promising and could be important for implementing these kinds of psycho-educational interventions within the school programmes in the classrooms along the academic year to improve children emotional skills.

## 5. Conclusions

The first results of the current study provide more evidence for the experimental program’s fundamental efficacy. Primary schoolchildren who participated in the experimental group showed more positive changes than controls on a measure of emotional recognition after the first year of intervention. Improvements in these specific domains are important in terms of the social, behavioural, and academic outcomes. Moreover, they could predict important life outcomes in adulthood and play a critical role in the behaviour change process [62]. Group intervention could be crucial to designing the role of schools in ensuring pupil emotional health and well-being, allowing children to develop their cooperative skills and achieve greater success both in school and in the future.

## Figures and Tables

**Table 1:** Mean (SD) of total scale, by age and group.

	Experimental Group			Control Group		
	(N = 116)			(N = 84)		
	Total	Male	Female	Total	Male	Female
Age	7.22 (0.97)	7.33 (1.04)	7.11 (0.9)	7.22 (0.93)	7.41 (1.03)	7.03 (0.83)
Danva-Pos Tot	7.59 (2.82)	7.33 (2.83)	7.85 (2.82)	8.61 (2.54)	8.65 (2.97)	8.58 (2.12)
Pofa	7.15 (1.57)	7.33 (1.54)	6.97 (1.60)	7.76 (1.89)	7.97 (1.47)	7.56 (2.31)
Classroom Drawing	0.95 (1.17)	0.86 (1.08)	1.07 (1.27)	1.66 (1.23)	1.77 (1.2)	1.55 (1.26)
MPC-TOT	67.13 (29.29)	69.16(29.43)	65.10 (29.16)	78.96 (20.3)	85(16.52)	72.92 (24.08)

Data are expressed as Mean (SD).

**Table 2:** Scale scores for experimental and control groups at baseline (T1) and post-intervention follow-up (T2).

	Experimental Group (N = 116)		Control Group (N = 84)	
	T1 Mean (SD)	T2 Mean (SD)	T1 Mean (SD)	T2 Mean (SD)
Pofa	7.08 (1.55)	8.14 (1.91) *	7.88 (1.99)	7.95 (1.59)
Danva Pos	7.48 (2.98)	8.25 (2.88) *	8.22 (2.21)	8.76 (2.46) *
MPC-TOT	62.53 (29.07)	68.25 (26.68) **	71.20 (26.69)	77.93 (22.41) **
Class Drawing	1.05 (1.20)	1.16 (1.21)	1.67 (1.26)	1.35 (1.34)

** *p* < 0.01; * *p* < 0.05.

**Table 3:** Number (%) of children in each Drawn Stories Technique (DST) outcome at baseline (T1) and post-intervention follow-up (T2).

Drawn Stories Technique	Experimental Group (N = 116)		Control Group (N = 84)	
	T1 No. (%)	T2 No. (%)	T1 No. (%)	T2 No. (%)
Positive Outcome	39 (33.6%)	40 (34.5%)	35 (41.7%)	42 (50%)
Negative Outcome	11 (9.4%)	13 (11.2%)	4 (4.7%)	11 (13.1%)
Positive Outcome Compensated	64 (55.2%)	60 (51,7%)	45 (53.5%)	30 (35.7%)
Absence Outcome	2 (1.7%)	3 (2.5%)	0 (0%)	1 (1.1%)

## Data Availability

Data are available upon request due to privacy restrictions.
